# Early White Matter Microstructure Alterations in Infants with Down Syndrome

**DOI:** 10.1016/j.neuroimage.2025.121489

**Published:** 2025-09-30

**Authors:** Omar Azrak, Dea Garic, Aleeshah Nasir, Meghan R. Swanson, Rebecca L. Grzadzinski, Khalid Al-Ali, Mark D. Shen, Jessica B. Girault, Tanya St. John, Juhi Pandey, Lonnie Zwaigenbaum, Annette M. Estes, Jason J. Wolff, Stephen R. Dager, Robert T. Schultz, Alan C. Evans, Jed T. Elison, Essa Yacoub, Sun Hyung Kim, Robert C. McKinstry, Guido Gerig, John R. Pruett, Joseph Piven, Kelly N. Botteron, Heather C. Hazlett, Natasha Marrus, Martin A. Styner

**Affiliations:** a Department of Psychiatry, University of North Carolina at Chapel Hill School of Medicine, Chapel Hill, NC, USA; b Carolina Institute for Developmental Disabilities, University of North Carolina at Chapel Hill School of Medicine, Chapel Hill, NC, USA; c Department of Pediatrics, University of Minnesota, Minneapolis, MN, USA; d Department of Psychiatry, Indiana University School of Medicine, Indianapolis, IN, USA; e University of Washington Autism Center, University of Washington, Seattle, WA, USA; f Center for Autism Research, Children’s Hospital of Philadelphia, University of Pennsylvania Perelman School of Medicine, Philadelphia, PA, USA; g Autism Research Centre, Department of Pediatrics, University of Alberta, Edmonton, Canada; h Center on Human Development and Disability, University of Washington, Seattle, WA, USA; i McConnell Brain Imaging Centre, Montreal Neurological Institute, McGill University, Montréal, Quebec, Canada; j Institute of Child Development, University of Minnesota, Minneapolis, MN, USA; k Department of Radiology, University of Minnesota, Minneapolis, MN, USA; l Mallinckrodt Institute of Radiology, Washington University School of Medicine, St. Louis, MO, US; m Tandon School of Engineering, New York University, New York, NY, USA; n Department of Psychiatry, Washington University School of Medicine, St. Louis, MO, USA

**Keywords:** Down syndrome, White matter tracts, Diffusion tensor imaging, Neurite orientation dispersion and density imaging, MRI, White matter microstructure

## Abstract

Down syndrome (DS), resulting from Trisomy 21, is the most prevalent chromosomal disorder and a leading cause of intellectual disability. Despite the significant impact of Trisomy 21 on brain development, research on white matter (WM) microstructure in infants with DS remains limited. While widespread reductions in WM integrity have been identified in children and young adults with DS, no study has examined WM microstructure in infancy. This study investigates early WM microstructure in infants with DS using diffusion tensor imaging (DTI) and neurite orientation dispersion and density imaging (NODDI). Forty-nine infants with DS (28 [57.14%] female) and 36 control (18 [48.65%] female) infants were scanned at 6 months of age. Infants with DS showed significant reductions in fractional anisotropy and neurite density index across multiple association tracts, particularly in the inferior fronto-occipital fasciculus and superior longitudinal fasciculus II, consistent with reduced structural integrity and neurite density. Increased radial diffusivity was observed in these tracts, a feature associated with disrupted myelination. In the inferior fronto-occipital fasciculus, superior longitudinal fasciculus II, and uncinate fasciculus, an elevated orientation dispersion index suggested increased neurite dispersion and fanning in infants with DS. These findings reveal widespread WM developmental alterations in DS, providing new insights into the early neurodevelopment of DS, which may inform timing of early therapeutic interventions.

## 1. Introduction

Down syndrome (DS), caused by Trisomy 21, is the most common genetic cause of intellectual disability, affecting approximately 1 in 600 newborns (Mai et al., 2019; Stallings et al., 2024). It is a neurogenetic syndrome characterized by developmental delays starting in infancy (Onnivello et al., 2023; Sacco et al., 2022), susceptibility to regression in childhood, and increased incidence of Alzheimer’s disease (AlzD) in adulthood (Gardiner et al., 2010; Rubenstein et al., 2024; Walpert et al., 2021). Neuroimaging research in DS has primarily focused on older children and adults, consistently implicating volumetric brain reductions and white matter (WM) integrity alterations in association with cognitive and functional challenges (Fenoll et al., 2017; Grzadzinski et al., 2024; Powell et al., 2014; Saini et al., 2022), even before the onset of AlzD. While previous studies in infants with DS have examined brain volume (Fukami-Gartner et al., 2023; Knickmeyer et al., 2008; McCann et al., 2021), no research has investigated WM microstructure at this early stage.

Infancy is a crucial period of brain development, particularly relative to WM maturation, which is marked by rapid myelination, heightened plasticity, and the elaboration of neural pathways subserving structural connectivity between brain regions (Grotheer et al., 2022; Kostović et al., 2019). In the case of DS, delineating differences in WM among infants with DS can clarify how Trisomy 21 influences brain and behavioral development, while also (a) providing insight into the emergence of atypical neurodevelopmental trajectories, (b) establishing a foundation for interpreting relationships of these trajectories to cognitive and functional outcomes, and (c) identifying optimal windows for mechanistically-informed interventions aimed at improving long-term cognitive and adaptive functioning in DS (Gao et al., 2015; Hamner et al., 2018).

Diffusion tensor imaging (DTI) is an MRI technique that models the diffusion of water molecules in tissue, capturing directional dependence (anisotropy) of diffusion due to microstructural barriers such as cell membranes and myelin, and thereby enabling detailed mapping of WM structures (Basser et al., 1994; O’Donnell & Westin, 2011). DTI has been instrumental in identifying WM abnormalities such as changes in fiber integrity and microstructural disruptions associated with demyelination and axonal damage (Baburamani et al., 2019; Basser & Pierpaoli, 1996; D. K. Jones & Cercignani, 2010), as well as tracking early brain development (Stephens et al., 2020; Wolff et al., 2012). However, DTI assumes a single dominant fiber orientation per voxel, making it difficult to interpret in regions with intersecting fibers (Guo et al., 2021). Neurite Orientation Dispersion and Density Imaging (NODDI) can address this challenge to obtain a more nuanced view of WM microstructure. NODDI is a diffusion MRI technique that models brain tissue as a combination of intra-cellular, extra-cellular, and CSF compartments, enabling quantification of neurite density and orientation dispersion—two microstructural properties that more specifically reflect underlying dendritic and axonal complexity than conventional DTI metrics (Zhang et al., 2012). Table 1 outlines DTI and NODDI parameters and their interpretations.

In children and young adults with DS, WM microstructural abnormalities, generally consistent with reduced structural integrity, have been identified in key tracts, including the inferior fronto-occipital fasciculus (IFOF), inferior longitudinal fasciculus (ILF), superior longitudinal fasciculus (SLF), corticospinal tract (CST), and uncinate fasciculus (UNC) (Garic et al., 2025; Gunbey et al., 2017; Romano et al., 2018). Here, we describe findings for diffusion MRI acquired in 6-month-old infants scanned during natural sleep as part of the Infant Brain Imaging Study of Down syndrome (IBIS-DS), a multisite prospective study of infant brain development in DS. We employed conventional DTI as well as Neurite Orientation Dispersion and Density Imaging (NODDI) to provide complementary metrics that allow a clearer interpretation of WM microstructure in regions with intersecting fibers (Guo et al., 2021). We hypothesized that infants with DS, similar to older children with DS, would exhibit lower fractional anisotropy (FA) and altered diffusivity in these tracts, reflecting atypical maturation of neural pathways critical for cognitive and motor function. By utilizing advanced, comprehensive neuroimaging methods, the study aims to make a first attempt to characterize WM microstructure in infants with DS, a critical step in understanding the neurodevelopment of DS from its earliest stages.

## 2. Methods

### 2.1. Participants

Neuroimaging data were collected in infants with DS during their 6-month study visit as part of the Infant Brain Imaging Study of Down syndrome (IBIS-DS) study led by the IBIS Network, which has conducted infant developmental neuroimaging studies for neurodevelopmental conditions including autism and Down syndrome. Infants with DS were reported by parents to have a diagnosis of full Trisomy 21 (i.e., not partial or mosaic Trisomy 21). Control infants, who did not have DS, were recruited within IBIS-DS and the IBIS Early Prediction Study (IBIS-EP), a prospective study of infants with an elevated likelihood of autism due to having an older sibling with autism. Controls recruited within IBIS-DS had no siblings or first-degree relatives with a history of autism or intellectual disability. Controls from IBIS-EP studies were confirmed to not meet clinical best estimate criteria for an autism diagnosis at age 24 months, based on the DSM-5 (American Psychiatric Association, 2013). Because of the study’s focus on characterizing a representative range of neurodevelopment in DS, a greater number of infants with DS were recruited than control infants, who provided a typically developing reference group. Analyses of homogeneity of variance confirmed that differing sample sizes between groups did not result in biased samples requiring additional corrections for analysis. Strict one-to-one matching was not implemented for analyses, although control participants were recruited concurrently and selected to be as similar as possible to the DS group in terms of age, sex, and socioeconomic status (SES). Supplementary Figure 1A details the number of scans initially collected, and how many were excluded during data processing.

Ethical approval was obtained from institutional review boards at all sites that relied on parent IRBs at Washington University in St. Louis, and written informed consent was obtained from each participant’s parent. Background on the IBIS infant studies and detailed exclusion criteria are available in supplementary methods.

### 2.2. MRI Acquisition and preprocessing

MRI scans were acquired during natural sleep on identical Siemens Prisma 3T scanners with a 32-channel head coil. Diffusion-weighted images (DWIs) were acquired in anterior-posterior (AP) and posterior-anterior (PA) phase-encoding directions, with 102 DWI volumes acquired for each AP and PA: 8 b=0, 20 b= 400, 37 b=1500, 37 b=3000, TR= 3222ms, TE=89.20ms, 1.5mm^3^ voxel, TA=12min19s. Tensors metrics were fit utilizing only b=400 and 1500 shells, while NODDI metrics were fit utilizing all available shells.

### 2.3. MRI Processing and Quality Control

All native data underwent automated quality control (QC) using DTIPrep1.2.9 (Oguz et al., 2014) to remove any poor-quality DWI volumes. A preliminary visual QC using 3D Slicer 5.1.0 was then performed by a trained rater who visually assessed the quality of the remaining volumes, excluding any scan with significant motion artifact, consistent signal dropout, or surviving volumes below a predetermined threshold (70%). For participants with multiple series, the rater selected the highest-quality series.

Following automated and preliminary visual QC, the remaining volumes were corrected for susceptibility, motion, and eddy current artifacts jointly via topup and eddy_openmp in FSL 6.0.3 (Andersson et al., 2017). In the same procedure, the remaining artifact/outlier data were corrected/interpolated in FSL 6.0.3 (Andersson et al., 2016). Brain masks were automatically generated as part of this procedure as these are necessary for the FSL processing. The automatic brain masks were computed via FSL bet applied to the (susceptibility corrected) average baseline/B0 image. All brain masks were reviewed and edited by trained raters.

Lastly, a final visual QC was conducted by a trained rater who assessed the corrected DWI volumes using DMRIPrep 0.5.0. During final visual QC, the rater inspected the individual corrected DWI volumes excluding any volumes with significant motion artifacts or signal dropouts. Concurrently, the rater evaluated each scan’s tractography based on the general expected anatomy, tract symmetry, and major tract integrity. Only scans with expected tractography and >80% surviving gradients passed. Scan-motion quantification was also computed during this step and is defined as the sum of diffusion volumes excluded during visual QC due to significant artifacts and those with head motion greater than 2 mm.

### 2.4. Atlas Creation, Mapping and Susceptibility Artifact Removal

A study-specific diffeomorphic, unbiased DTI atlas was created using the UNC-Utah National Alliance for Medical Image Computing DTI framework (Verde et al., 2014). Brain-masked Diffusion Tensor Images (weighted least squares estimation of tensors) of 70 subjects were used to create the atlas. Then, each individual subject DTI were mapped to atlas space using nonlinear, diffeomorphic pair-wise registration, with visual inspection to verify registration accuracy.

A susceptibility artifact in the AP phase affecting the temporal poles was found in 50 scans (Supplementary Figure 2). To remove the artifact from the analyses, the region containing the artifact was manually segmented in 10 individual DWI scans where the artifact appeared most prominent, focusing on volumes where it showed the highest intensity. The resulting mask was then propagated to the DTI atlas for the entire sample. The affected tracts included the tapetum of the corpus callosum, the bilateral ILFs, fornices, and UNCs. There was no significant group difference in the occurrence rate of the artifact (*X*^2^=1.44, *p*=0.23).

### 2.5. Tractography and WM Metric Extraction

Metrics of WM microstructure were extracted via an extended UNC-NAMIC automated fiber analysis framework (Verde et al., 2014). Fiber tractography was performed using AutoTract, an atlas-guided tractography tool that generates subject-specific tracts by propagating predefined atlas fiber labels through nonlinear registration, followed by fiber clustering to extract anatomically consistent bundles in individual scans (Prieto et al., 2016). Tracking was carried out on individual DTI scans in atlas space. Manual refinement was then performed by a trained editor using 3D Slicer 5.1.0, involving visual inspection and editing of the resulting tracts according to established anatomical definitions (Short et al., 2022). The resulting deformation fields mapped atlas fibers to individual subject spaces, where diffusion tensor and NODDI metrics were extracted at equidistant points along each fiber tract. Diffusion tensors were estimated via a weighted least squares approach in Dmriprep (Dubos et al., 2023), DTI properties—FA, axial diffusivity (AD), radial diffusivity (RD), mean diffusivity (MD)—were extracted from the estimated tensors. NODDI properties—neurite density index (NDI) and orientation dispersion index (ODI)—were generated using Accelerated Microstructure Imaging via Convex Optimization (AMICO) (Daducci et al. 2015).

For quality control, we excluded subjects whose tract-specific FA profiles correlated poorly (r < 0.70) with the population average, as this typically indicates suboptimal DTI-to-atlas alignment. Final tract metrics were computed by averaging the respective diffusion parameters along each fiber tract.

**Figure 1.**
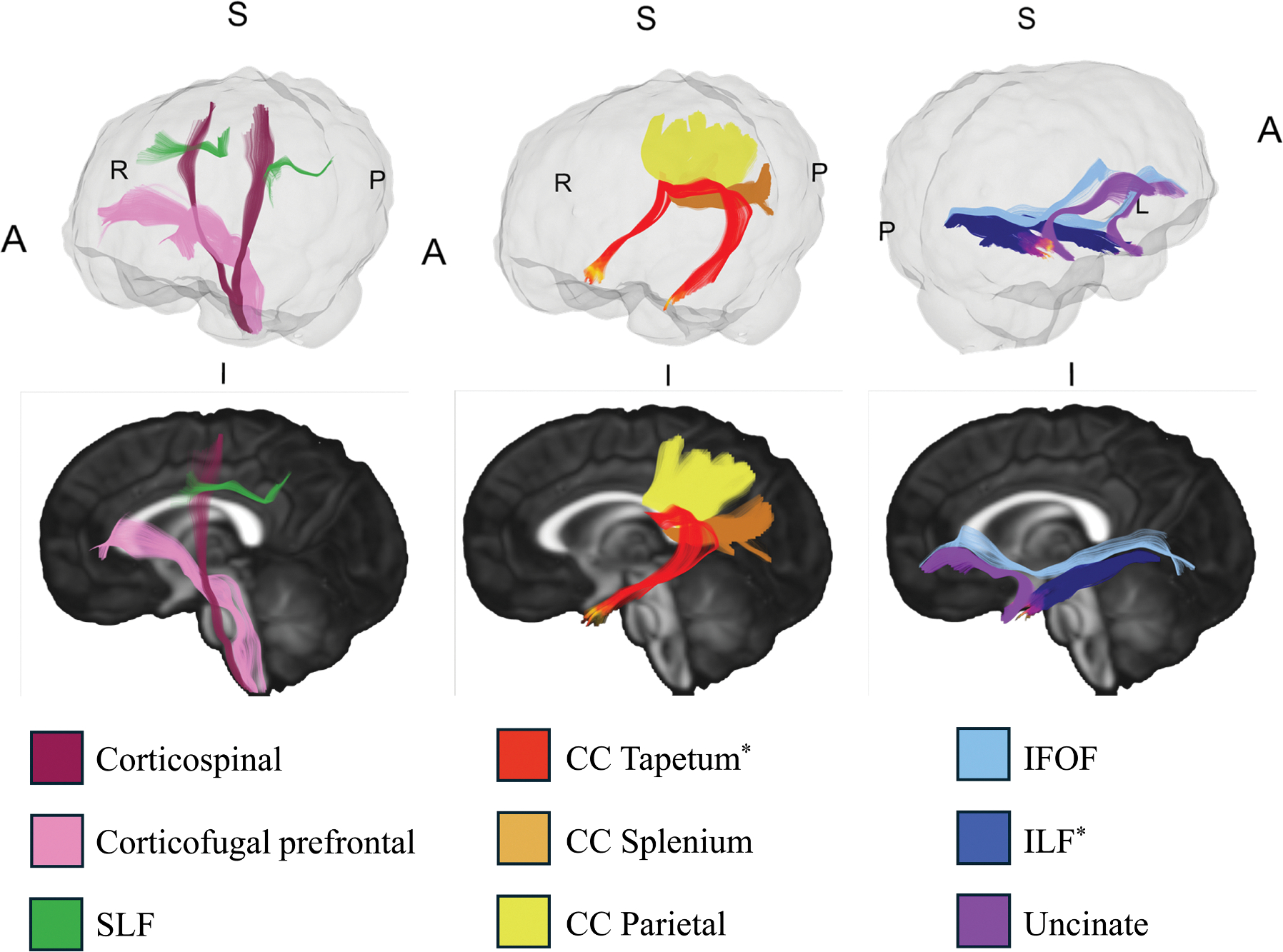
Fiber tractography of the examined tracts. Tracts marked with an asterisk (*) were affected by the susceptibility artifact; affected segments are color-coded with distinct warm colors to distinguish them from the rest of the tract. Abbreviations: IFOF, inferior fronto-occipital fasciculus; ILF, inferior longitudinal fasciculus; SLF, superior longitudinal fasciculus; CC, corpus callosum; A, anterior; P, posterior; S, superior; I, inferior; R, right; L, left.

Drawing on previous findings in older children (Garic et al., 2025; Gunbey et al., 2017), we examined 6 intrahemispheric tracts bilaterally—corticofugal prefrontal, CST, IFOF, ILF, SLF II, and UNC—and 3 interhemispheric tracts: the parietal portion, splenium, and tapetum of the corpus callosum (CC). Intrahemispheric tracts—including the CST, SLF, and UNC—support motor, language, and memory functions, all of which are commonly affected in those with DS (Bazydlo et al., 2021; Beqaj et al., 2017; Brauer et al., 2013; Dick et al., 2014; Jain et al., 2022; Naigles et al., 2017; Powell et al., 2014; Samara et al., 2019;Zemmoura et al., 2021). For example, CST alterations have been linked to delays in motor milestones among adolescents and adults with DS (Beqaj et al., 2017; Fenoll et al., 2017; Frank and Esbensen, 2015; Jain et al., 2022; Jung et al., 2017; Romano et al., 2018). The selected interhemispheric tracts, such as the CC, have been associated with impaired interhemispheric communication and atypical language lateralization in this population (Heath et al., 2005; Piccirilli et al., 2023; Saini et al., 2022; Shoji et al., 2009). All primary analysis tracts, with the portions affected by the susceptibility artifact, are displayed in Figure 1.

Supplementary Figure 1B provides a visual flowchart of the complete data processing pipeline. All processing steps and the corresponding scripts used at each stage are outlined and available at https://github.com/ale-nasir/WMMA-Infants-DS-Pipeline.git.

### 2.6. Statistical Analyses

All tract average-based statistical analyses were performed using general linear models (GLMs) in JMP 17 Pro.

#### 2.6.1. Primary Analyses

For each tract, a multivariate analysis of variance (MANOVA) was conducted comparing the average values of FA, RD, AD, NDI, and ODI between the DS and control groups. MD was excluded to avoid multicollinearity with AD and RD in MANOVA, while FWF was excluded due to its sensitivity to partial volume effects, particularly in tracts close to the cerebrospinal fluid (CSF), like the CC. Results were reported using both Wilks’ Lambda and Pillai’s Trace, the latter of which is considered more robust in the presence of unequal sample sizes or mild violations of the homogeneity of covariance assumption.

For tracts showing significant group differences in MANOVA, follow-up univariate analyses of variance (ANOVA) were conducted to identify the specific diffusion parameters significantly different between the two groups. We tested the assumption of homogeneity of variance for each ANOVA and confirmed that the equal variance assumption was met for all DTI and NODDI measures (*p* > 0.05). All analyses were covaried by age-at-assessment in days (defined as postnatal age at scan), gestational age, sex, and scan-motion quantification. To correct for multiple comparisons, Bonferroni correction was applied across all tests, accounting for 15 comparisons in MANOVA (*corrected p*<*0.003*) and 5 diffusion parameter comparisons in ANOVA (*corrected p*<*0.01*). All reported *p*-*values* from ANOVA tests in the Results section 3.2 are corrected.

#### 2.6.2. Secondary Analyses

For tracts identified as significant in ANOVA, along-tract analyses were conducted using the Functional Analysis of Diffusion Tensor Tract Statistics (FADTTS) toolbox (Zhu et al., 2011) and its corresponding graphical user interface, FADTTSter (Noel et al., 2017). Along-tract analyses were performed for each diffusion parameter (FA, RD, AD, NDI, ODI) identified as significant in prior ANOVA tests, providing a finer-grained understanding of where differences occurred in the tract. A detailed process of the along-tract analysis is available in the supplementary material. Results were assessed visually and statistically to interpret region-specific differences in diffusion properties.

#### 2.6.3. Exploratory Analyses

Exploratory full-brain analyses were performed to examine all diffusion parameters (FA, AD, RD, MD, NDI, ODI, and FWF) across all 51 tracts (listed in Supplementary Table 1). MD and FWF were only evaluated within this exploratory context and are relevant to understanding WM microstructure. Effect sizes were provided without correcting for multiple comparisons to maximize detection of potential differences between groups that could inform future hypothesis generation.

## 3. Results

### 3.1. Demographics

A total of 49 infants with DS and 36 control infants were included. No significant differences were observed in sex (*X*^*2*^=0.43, *p*=0.51), age-at-assessment in days (*p*=0.13), and scan motion quantification (*p*=0.4). Gestational age was slightly lower in infants with DS (266 ± 9.7 days) than in control infants (273 ± 9 days; *p*=0.004). Maternal age at birth was also significantly higher in infants with DS (36.84 ± 4.54 years) than in control infants (34.19 ± 3.06 years; *p*=0.005), consistent with the increased incidence of DS with maternal age. A summary of the demographics can be found in Table 2. Demographics related to education and household income can be found in Supplementary Table 2.

### 3.2. Primary Analyses: Analysis of Variance of Diffusion Parameter Averages Across Tracts

Significant group differences were found between DS and control groups on MANOVA in the tapetum and parietal portions of the CC, as well as the right CST, and bilateral IFOF, ILF, SLF II, and UNC. The left SLF II (*F*=9.09, *p*<.0001), right SLF II (*F*=7.97, *p*<.0001), left IFOF (*F*=8.23, *p*<.0001) and parietal CC (*F*=6.83, *p*<.0001) showed the strongest statistical differences on MANOVA. The splenium of the CC and bilateral corticofugal prefrontal tracts showed no group differences, and the left CST did not retain significance after correction. Supplementary Table 3 details the MANOVA results.

On univariate analysis, several association tracts in infants with DS showed patterns consistent with reduced structural integrity and neurite density as suggested by reduced FA and NDI, as well as altered maturation and increased neurite dispersion or fanning as inferred by elevated RD and ODI.

The IFOF in particular showed bilateral reductions in all four of these diffusion metrics in infants with DS: FA (Left: *β*=0.00986, *p*<.0001; Right: *β*=0.00775, *p*=0.0005), NDI (Left: *β*=0.00529, *p*=0.012; Right: *β*=0.0065, *p*=0.0025), RD (Left: *β*=−0.0000115, *p*=0.001; Right: *β*=−0.0000168, *p*=0.001) and ODI (Left: β=−0.00527, p<.0001; Right: β=−0.00584, p=0.02).

Notably, the SLF II showed bilateral reductions in FA (Left: *β*=0.0104, *p*<.0001; Right: *β*=0.00685, *p*=0.032), NDI (Left: *β*=0.00737, *p*=0.0015; Right: *β*=0.00738, *p*=0.001), and RD (Left: *β*=−0.0000148, *p*=0.0005; Right: *β*=−0.00001, *p*=0.031), but significant reductions on the left only for ODI (β=−0.00746, p=0.002) in infants with DS.

Similarly, the UNC showed bilateral reductions in FA (Left: β=0.00531, p=0.03; Right: β=0.00695, p<.0001) but significant reductions on the right only for ODI (β=−0.00407, p=0.017) in infants with DS. Lastly, the ILF and parietal portion of the CC only showed reductions in NDI (Left ILF: β=0.00861, p=0.0035; Right ILF: β=0.00746, p=0.019; Parietal CC: β=0.006, p=0.017) in infants with DS.

**Figure 2.**
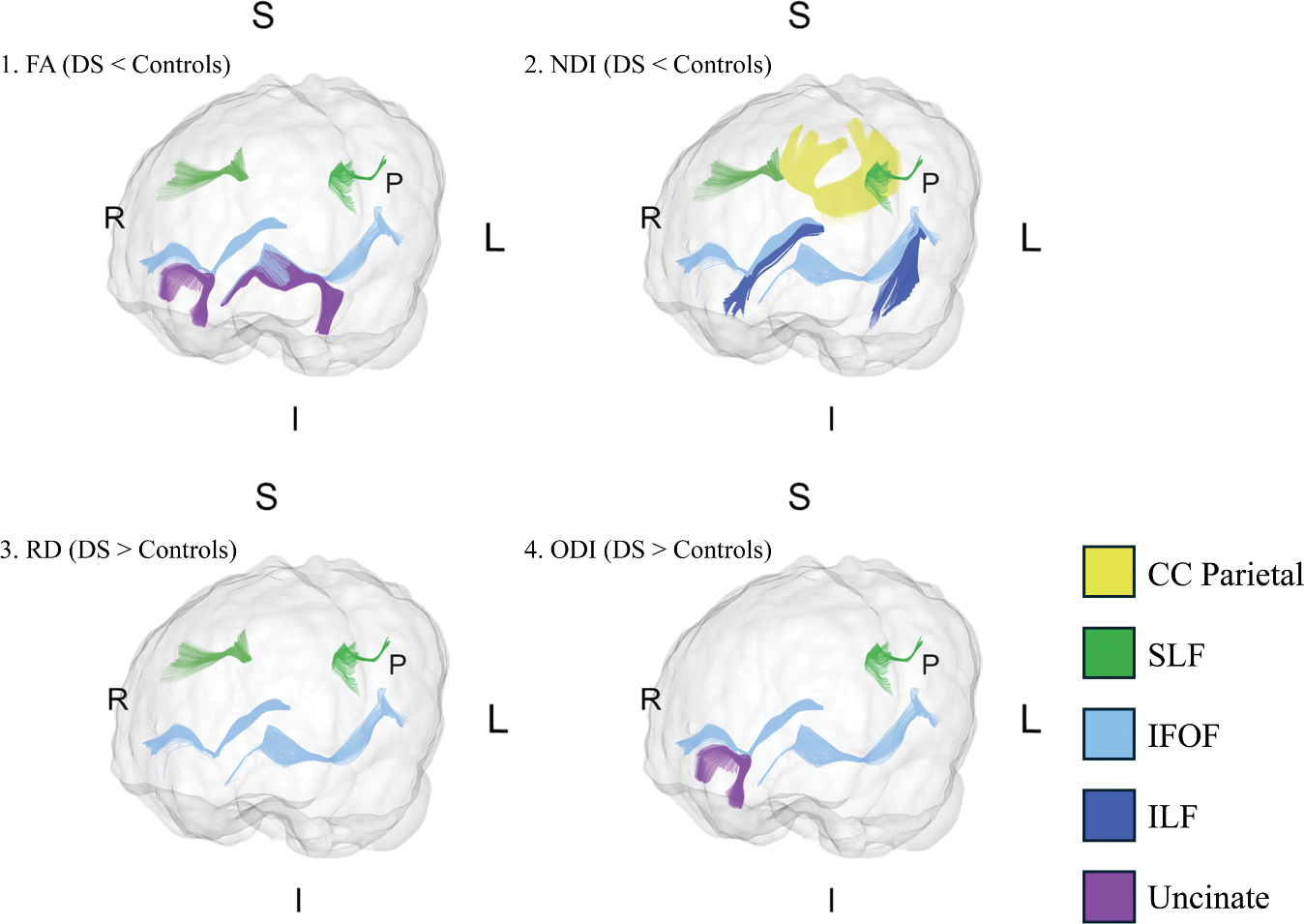
3D models of the tracts showing significant group differences for each diffusion parameter. For (1) FA and (2) NDI, all significant tracts exhibited lower values in infants with DS compared to control infants (DS < Controls). For (3) RD and (4) ODI, all significant tracts showed higher values in infants with DS compared to control infants (DS > Controls). Abbreviations: FA, fractional anisotropy; NDI, neurite density index; RD, radial diffusivity; ODI, orientation dispersion index; CC, corpus callosum; SLF, superior longitudinal fasciculus; IFOF, inferior fronto-occipital fasciculus; ILF, inferior longitudinal fasciculus; P, posterior; S, superior; I, inferior; R, right; L, left.

**Figure 3.**
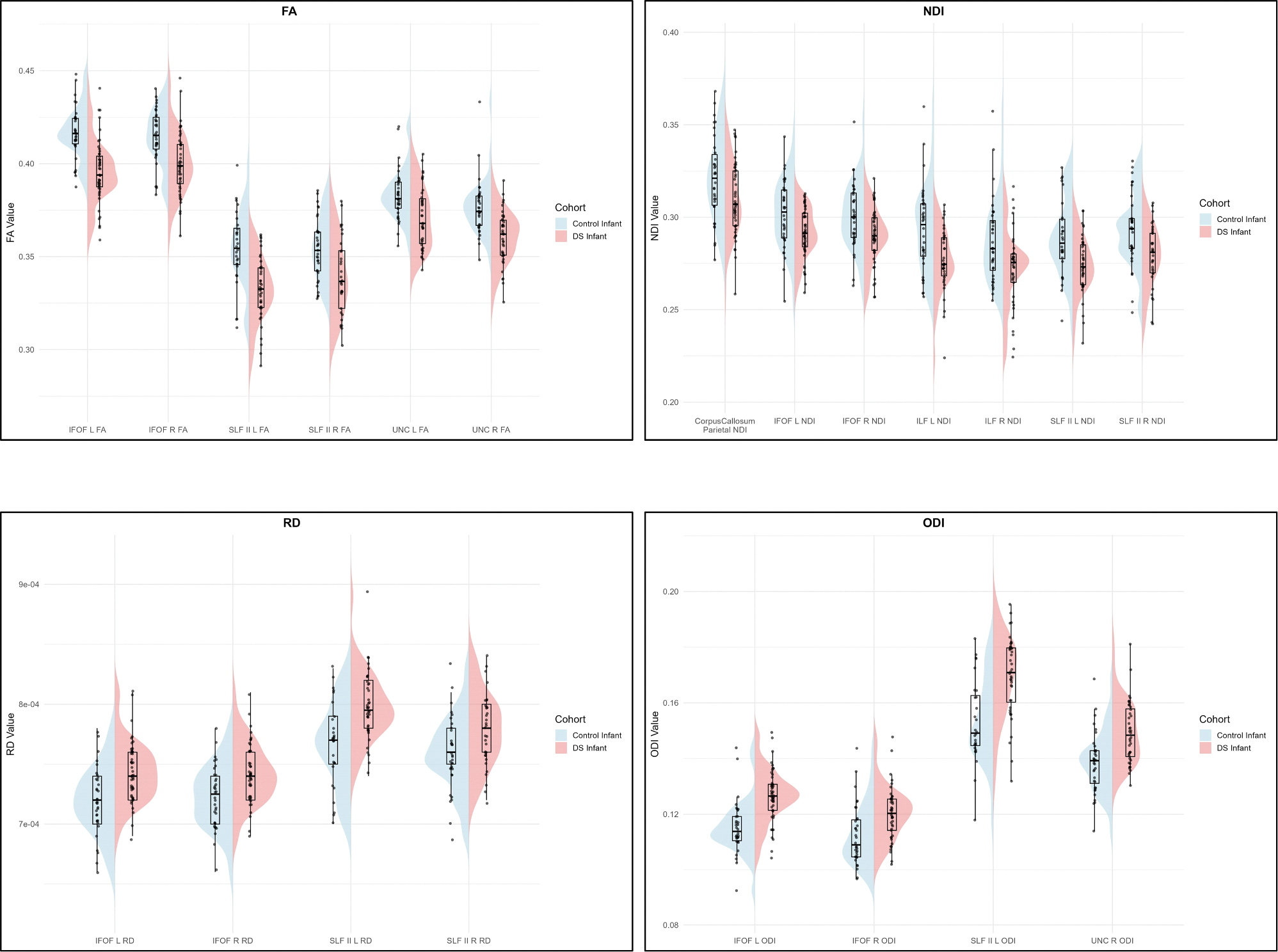
Violin and box plots of the significant diffusion metrics in all tracts. Abbreviations: IFOF, inferior fronto-occipital fasciculus; ILF, inferior longitudinal fasciculus; SLF, superior longitudinal fasciculus; UNC, uncinate fasciculus; FA, fractional anisotropy; RD, radial diffusivity; NDI, neurite density index; ODI, orientation dispersion index.

Effect sizes for significant tract metrics were moderate to large, with absolute Cohen’s d values ranging from 0.55 to 1.34. No tracts retained significant AD differences after correcting for multiple comparisons. The tapetum of the CC and the right CST also showed no significant differences after correcting for multiple comparisons. Figure 2 presents 3D models of the tracts showing significant group differences for each parameter (FA, RD, NDI, ODI). Figure 3 presents violin and box plots of the significant diffusion metrics. Table 3 details the ANOVA results for tracts found significant on MANOVA.

### 3.3. Secondary Analyses: Along-Tract Differences

**Figure 4.**
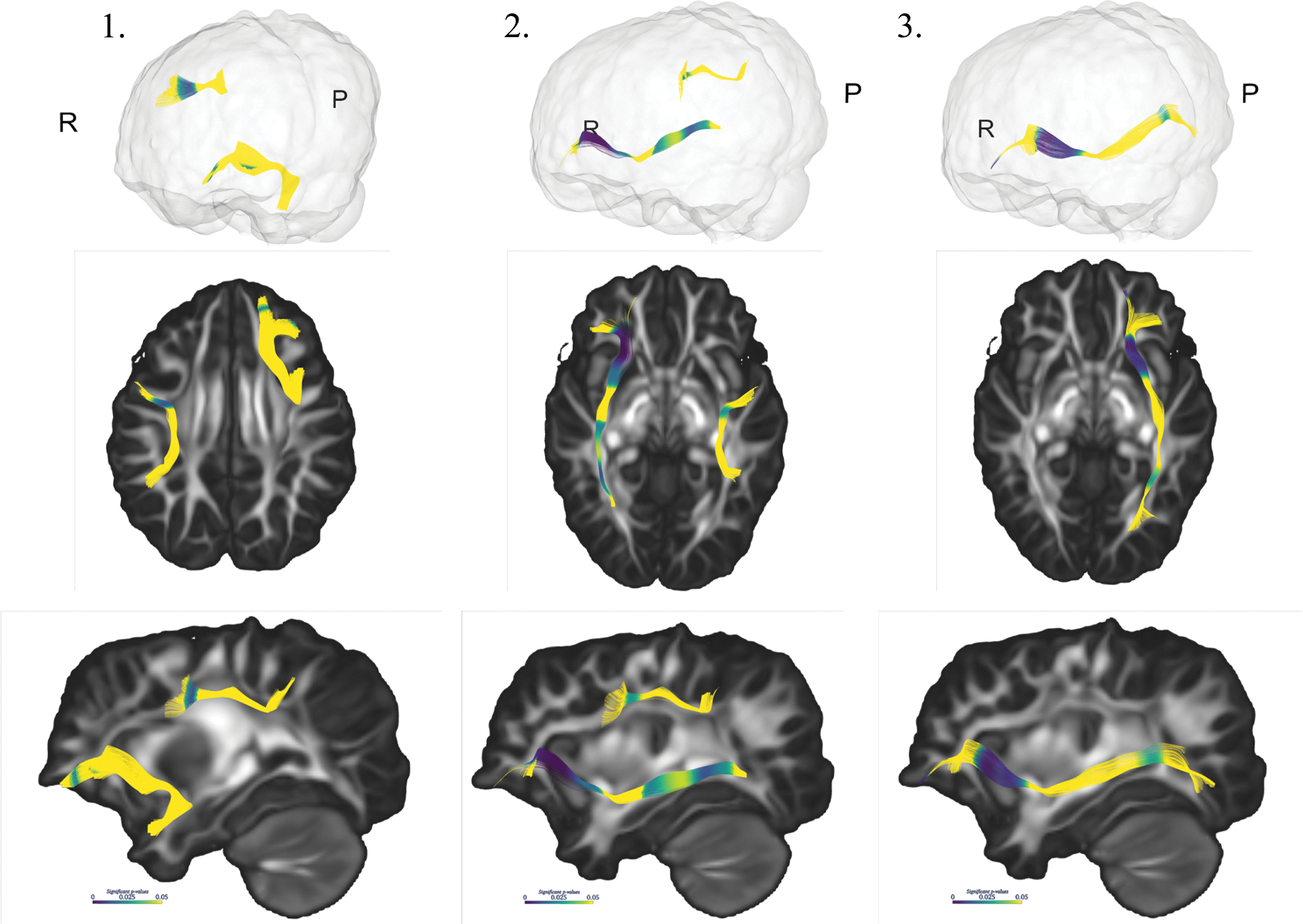
Visualization of the significant *p*-values along the tracts generated using 3D Slicer. 1. displays significant regions of FA in the right SLF II and the left UNC, 2. highlights significant NDI in the left SLF II and the right IFOF, and 3. shows significant NDI in the left IFOF. Abbreviations: IFOF, inferior fronto-occipital fasciculus; SLF, superior longitudinal fasciculus; UNC, uncinate fasciculus; FA, fractional anisotropy; NDI, neurite density index.

We conducted follow-up along-tract analyses for the diffusion parameters found significant in ANOVA (CC Parietal, IFOF, SLF II, ILF, and UNC) to identify spatially-specific differences along the trajectories of fiber tracts. Along-tract analysis of the bilateral IFOFs demonstrated differences in NDI and RD across the frontal, temporal and parietal portions of the tracts, with the most pronounced differences observed in NDI. In addition, the right IFOF exhibited FA differences in its parietal portion. The bilateral SLF II demonstrated alterations in the ventral segments of the tracts as they extend into the frontal lobes, involving NDI and RD in the left SLF II and FA of the right SLF II. Lastly, the left UNC exhibited differences in FA in its frontal portion. Figure 4 visualizes the significant *p*-values along the tracts.

### 3.4. Exploratory Analyses: Full-brain analysis

Exploratory full-brain analyses across all 51 tracts revealed distinct patterns in diffusion parameters between infants with DS and control infants. No correction for multiple comparisons was applied for the exploratory analyses. Full statistical results across all tracts are presented in Supplementary Table 4.

FA values were lower in infants with DS across all significant tracts except for the bilateral optic tracts, which exhibited higher FA values. MD values were consistently higher in infants with DS, e.g., in the left frontoparietal arcuate fasciculus (*p*=0.0049), left SLF II (*p*=0.0022), and several portions of the left corticothalamic tract (Motor: *p*=0.0003; superior: *p*=0.0001). RD values were higher in infants with DS, with the exception of the right optic tract and AD was only significantly lower for the left cingulate gyrus of the cingulum (CGC) while it was significantly higher for many tracts such as the left fornix and left optic radiation. Significant *p*-values (uncorrected) ranged from 0.0001 to 0.0451 for AD, and from 0.0001 to 0.0457 for RD.

NDI values were consistently lower in infants with DS across all significant tracts. Examples include several portions of the CC (Body: *p*<.0001; Motor: *p*<.0001; Parietal: *p*=0.0034), bilateral motor portions of the corticothalamic tract *(p*<.0001), and the premotor portion of the right corticothalamic tract *(p*=0.0012). ODI values varied across tracts, with some showing increases and others decreases in infants with DS (Significant *p*-values ranged from <.0001 to 0.049). FWF values were lower in infants with DS in all significant tracts (CC Body: *p*<.0001; Motor CC: *p*=0.0005; Parietal CC: *p*<.0001; Bilateral motor corticothalamic: *p*<.0001; right premotor corticothalamic: *p*=0.0001) except for the CST bilaterally and the right hippocampal part of the cingulum (HCG).

Notably, the optic tracts were the only tracts to demonstrate higher FA bilaterally (*p*<.0001), higher AD value in the left (*p*=0.0014) and lower RD in the right (*p*=0.0003) alongside lower ODI values bilaterally (*p*<.0001) in infants with DS, which could correspond to a greater maturation or preservation of WM microstructure for this tract in infants with DS.

## 4. Discussion

### 4.1. FA and RD: Microstructural Organization and Regional Variability

This is the first study to examine the WM microstructure in infants with DS. Relative to control infants without DS, differences across several diffusion metrics were consistent with signs of atypical WM maturation, including regional differences in neurite density, axonal growth, and myelination across multiple tracts. Findings were also consistent with reduced microstructural coherence and disrupted myelination, as evidenced by lower FA and elevated RD in intrahemispheric tracts such as the bilateral IFOF and SLF, and lower FA in the UNC in infants with DS. These results align with prior DS research demonstrating WM differences in early childhood to adulthood (Garic et al., 2025; Gunbey et al., 2017; Romano et al., 2018; Saini et al., 2022) and suggest that some of the microstructural differences observed at infancy may represent early-emerging features of a lifelong trajectory of disrupted WM in DS.

### 4.2. NDI: Evidence for Higher Sensitivity in Detecting Neurite Growth Disruptions

Previous studies suggest that NDI typically reflects neurite density, which generally increases during the first two decades of life (Kunz et al., 2014; Zhang et al., 2012). NDI was significantly reduced in infants with DS across most major WM tracts, including the SLF II, IFOF, ILF, and the parietal portion of the CC. Lower NDI in infants with DS thus implies a slower rate of neurite packing, particularly in axons and dendrites, during this critical developmental window. Consistent with Timmers et al. (2016), NDI demonstrated greater sensitivity, identifying more tracts with lower values compared to FA. Combined with reductions in FA, the lower NDI provides converging evidence of global disruptions in WM maturation in infants with DS.

### 4.3. ODI: Evidence of Compensatory Reorganization

Findings for ODI, which corresponds to the degree of alignment in neurite orientation, further highlight regional variability in neurite structure. In infants with DS, higher ODI in the IFOF and right UNC suggests greater neurite dispersion, corresponding to findings by Garic et al. (2025) in school-aged children. Taken together with the NDI results, higher ODI may reflect compensatory reorganization, such as axonal sprouting or disrupted pruning, in response to reduced neurite density suggested by lower NDI (Radetz et al., 2021; Timmers et al., 2015).

### 4.4. Along-tract Analyses: Spatial Analysis of the WM Tracts in DS

To complement whole-tract averages, we employed along-tract analyses, which provided a spatially specific assessment of WM changes (Rieck et al., 2020; Shirazi et al., 2021). Prior studies have shown that averaging diffusion metrics across an entire tract can obscure important localized differences (Colby et al., 2012; Yeatman et al., 2012). In our study, the along-tract approach enabled us to identify localized alterations within WM segments generally associated with behavioral domains affected in DS. For instance, in infants with DS, reduced FA and NDI and elevated RD were observed in the frontal portion of the SLF II, a segment implicated in expressive language and executive function (Brauer et al., 2013; Dick et al., 2014). Additionally, the IFOF, a tract known for its involvement in long-range semantic and socio-emotional integration (Panesar et al., 2018; Sarubbo et al., 2013), showed significantly reduced FA, reduced NDI, and increased RD across its frontal, parietal, and temporal segments. Lastly, in the frontal segment of the left UNC, FA was reduced, which may reflect early disorganization in a tract associated with emotion regulation and memory (Olson et al., 2015; Von Der Heide et al., 2013). These findings reveal anatomically specific group differences that offer a foundation for future studies to investigate region-specific WM maturation and explore the biological and behavioral implications of localized diffusion changes—particularly given the limited literature linking along-tract profiles to developmental processes in infancy and in DS.

The absence of significant along-tract findings in other tracts, despite group differences detected in average parameter analyses, could be attributed to the increased number of comparisons required by along-tract approaches, which demand a larger sample size to detect significant localized changes along the tracts (Yeatman et al., 2012; Zhu et al., 2011).

### 4.5. AD: Evidence of Alterations in Axial Development in Exploratory Full-brain Analysis

In exploratory analysis, noticeable patterns of AD were observed across tracts suggesting region-specific alterations in axonal organization in infants with DS. For example, significantly lower AD alongside reduced FA and elevated RD in the left cingulum cingulate (CGC) highlights the early vulnerability of a pathway associated with attention, memory and emotional regulation (Kubicki et al., 2009; Nestor et al., 2004;Posner et al., 2007; Saini et al., 2022). However, elevated AD was observed in other regions, such as the left fornix and left optic radiation. Although decreased AD is often interpreted as indicative of disruptions in axonal organization, some studies have reported increased AD in the context of neurodegenerative risk. Romano et al. (2018), for example, observed elevated AD in adolescents and young adults with DS and noted that such increases may represent early microstructural changes related to susceptibility to AlzD, although not necessarily degeneration. The presence of elevated AD in infancy may therefore reflect early alterations in axonal geometry or extracellular water content, warranting further longitudinal investigation to clarify its developmental and clinical significance.

### 4.6. MD: Patterns of Disrupted Myelination in Exploratory Full-brain Analysis

Exploratory analyses showed higher MD in specific tracts in infants with DS, including the frontoparietal portion of the left arcuate fasciculus, left SLF II, and left corticothalamic motor and superior pathways. In typical development, myelination progresses posterior-to-anterior and caudal-to-rostral, with occipital/parietal lobes myelination occurring between 4–6 months and frontal/temporal lobes between 6–8 months (Brody et al., 1987; S. Deoni et al., 2018; S. C. L. Deoni et al., 2011; Suzuki, 2007). Reductions in MD are therefore anticipated as part of typical neurodevelopment around age 6 months. The observation that infants with DS exhibited higher MD in these tracts, compared to controls of the same age, could suggest a disrupted timeline of myelination in infants with DS, with slower and less robust myelination.

### 4.7. FWF Reductions Suggest Absence of Neuroinflammation in Exploratory Full-brain Analysis

In our exploratory analyses, significant reductions in FWF were observed in the CC (body, motor, parietal) and corticothalamic motor and right premotor tracts, which also showed concurrent reductions in NDI. This pattern suggests reduced neurite density without evidence of neuroinflammation. Increased FWF has been documented in older adults with AlzD (Dumont et al., 2019; Ji et al., 2017), a neurodegenerative condition associated with early onset in DS, purportedly due to the triplicated amyloid precursor protein on chromosome 21 (Bazydlo et al., 2021; Rubenstein et al., 2024; Salehi et al., 2015). The reduced FWF observed in infants with DS suggests an absence of inflammatory or degenerative processes at this stage, highlighting the need for longitudinal studies to delineate the progression of pathological changes in WM across the lifespan.

### 4.8. Optic Tracts: Unique Patterns in DS

Infants with DS exhibited higher FA in the optic tracts bilaterally, consistent with findings from Gunbey et al. (2017), who observed elevated FA in the right optic tract of two-year-old children with DS. Furthermore, reduced ODI in the optic tracts aligns with these findings, reflecting less dispersion and greater coherence in fiber orientation, suggesting an early maturation of the visual pathway relative to higher-order systems (Ford et al., 2023; W. Jones & Klin, 2013). However, this early structural advantage may not translate to functional gains over time, as studies show that visual acuity plateaus after two years in children with DS (Woodhouse et al., 1996), while it continues to improve in typically developing peers (Leat et al., 2009; McDONALD et al., 1986).

### 4.9. WM as an Early Biomarker and Therapeutic Target in DS Neurodevelopment

Our findings demonstrate that WM microstructural alterations emerge early in infants with DS, mirroring patterns observed in later stages (Bazydlo et al., 2021; Fenoll et al., 2017; Garic et al., 2025; Gunbey et al., 2017; Powell et al., 2014; Romano et al., 2018; Saini et al., 2022) and the early onset of developmental delays in DS. The observation of measurable differences in multiple tracts underscores the importance of exploring this underrepresented field in DS neurodevelopment. Early interventions and therapies have been shown to improve outcomes in individuals with DS (Hendrix et al., 2021), and WM may serve as a valuable biomarker for monitoring these interventions, as it has shown to change in response to treatment in children and adults (Swanson & Hazlett, 2019). Notably, a prior clinical trial reported improvements in WM connectivity following treatment in children with autism (Carpenter et al., 2019). This study represents a crucial foundation for understanding the neurobiology of DS and identifying early WM alterations that may inform optimal windows for mechanistically-derived interventions aimed at improving long-term outcomes.

### 4.10. Strengths, Limitations and Future Directions

A key strength of our study is its larger sample of DS and control infants compared to previous studies in toddlers, the largest of which (Gunbey et al., 2017) included only 10 DS and 8 control individuals. The larger sample size enhances the reliability and generalizability of our findings. The use of multishell diffusion imaging allowed us to obtain and examine both DTI and NODDI data in infants with DS. NODDI distinguishes intra-neurite, extra-neurite, and CSF compartments, estimating neurite density and orientation dispersion (Collorone et al., 2020; Li et al., 2024; Zhang et al., 2012). These measures complement traditional DTI, helping disentangle the effects of axonal density, myelination, and neurite organization (Beaulieu, 2009; Friedrich et al., 2020).

In terms of limitations, future studies of WM microstructure in a larger sample of infants with DS would improve sensitivity to detect smaller effects in along-tract analyses, and an opportunity to test for replication of present findings, which is particularly relevant given growing recognition of heterogeneity within DS (Windsperger & Hoehl, 2021). Additionally, the observed susceptibility artifact, as described in section 2.4, necessitated a more restrictive definition of some tracts, which could have limited detection of differences in the affected tracts. Future research also warrants longitudinal analyses to contextualize WM microstructure differences relative to the course of neurodevelopment. Such analyses could clarify whether observed differences constitute a delay or divergence from typical development, or represent persistent or transient alterations, as well as test for association with brain alterations previously observed at later stages in individuals with DS. Within the IBIS study, an important future direction is to examine how WM microstructure in infancy correlates with behavioral, language, and cognition later in childhood, potentially providing a predictive tool for these developmental outcomes.

## 5. Conclusion

By employing advanced diffusion imaging techniques, this study provides evidence for alterations in multiple aspects of WM microstructure in infants with DS, addressing a significant gap in research that has largely focused on older children and adults. Our findings reveal patterns consistent with reduced myelination, lower neurite density, and increased neural dispersion in fibers critical for higher-order cognitive functions, providing an early window into the features of atypical neurodevelopment that may underlie cognitive and motor delays in DS. These observations lay a novel foundation for future longitudinal studies to explore how early WM alterations relate to cognitive, behavioral, and motor outcomes in DS, and will be essential for identifying critical windows for targeted clinical interventions aimed at supporting WM maturation and mitigating developmental delays.

## Supplementary Material

1

2

Supplementary materials

Supplementary material associated with this article can be found, in the online version, at doi:10.1016/j.neuroimage.2025.121489.

## Figures and Tables

**Table 1:** Overview of DTI and NODDI metrics with associated biological interpretations.

Metric^a^	Definition of Measurement	Interpretations of Measurements from prior studies

**DTI Metrics**		
Fractional Anisotropy (FA)	A scalar value between 0 and 1 that quantifies the degree of anisotropy of water diffusion; 0 indicates isotropic diffusion, and 1 indicates fully anisotropic diffusion (Basser & Pierpaoli, 1996; Rouine et al., 2018).	Decreased FA values suggest a delay/disruption in the structural integrity of white matter (Fenoll et al., 2017; Gunbey et al., 2017; Powell et al., 2014; Saini et al., 2022).
Axial Diffusivity (AD)	The magnitude of water diffusion parallel to the tract (Basser et al., 1994; Song et al., 2002; Winklewski et al., 2018).	Decreased AD suggests delayed/disrupted axonal organization and elongation (Aung et al., 2013; Budde et al., 2009; Nguyen et al., 2019).
Radial Diffusivity (RD)	The magnitude of water diffusion perpendicular to the tract (Basser et al., 1994; Song et al., 2002; Winklewski et al., 2018).	Increased RD suggests delayed/disrupted myelination (Keller & Just, 2009; Rosas et al., 2020; Song et al., 2002; Winklewski et al., 2018).
Mean Diffusivity (MD)	The average amount of diffusion occurring within a single voxel (Basser et al., 1994; Basser & Pierpaoli, 1996; Gunbey et al., 2017).	Increased MD suggests delayed/disrupted tissue organization (Fleming et al., 2021; Narr et al., 2009; Saini et al., 2022).
**NODDI Metrics**		
Neurite Density Index (NDI)	Reflects the fraction of tissue volume occupied by neurites (axons and dendrites) (Kraguljac et al., 2023; Zhang et al., 2012).	Decreased NDI suggests a delayed/disruption in neurite density (Andica et al., 2021; Arai et al., 2023).
Orientation Dispersion Index (ODI)	The variability in neurite orientation, ranging from 0 (perfectly aligned) to 1 (randomly in all directions) (Kraguljac et al., 2023; Zhang et al., 2012).	Increased ODI suggests greater dispersion and fanning of neurites (Chang et al., 2015; Matsuoka et al., 2020; Zhang et al., 2012).
Free Water Fraction (FWF)	The fraction of diffusion signals explained by isotropically unrestricted water, estimated using a bi-tensor model (Pasternak et al., 2009).	Increased FWF suggests neuroinflammation (Nakaya et al., 2022; Takeshige-Amano et al., 2024).

a FA, NDI, ODI, and FWF are unitless values representing a range in these respective properties between 0 and 1. AD, RD, and MD are reported in mm^2^/s.

**Table 2:** Participant demographics by group.

	Down syndrome (DS)	Controls	Group Comparison

**N**	49	36	
**Sex, n (%)**	Female: 28 (57.14) Male: 21 (42.86)	Female: 18 (50) Male: 18 (50)	*X^2^*=0.426, *p*-value=0.5138
**Age-at-assessment in days**^a^**, Mean (SD)** **(Range)**	207.31 (25.10) (176–297)	199.67 (19.36) (175–258)	t-test, *p*-value=0.1317
**Gestational Age at birth in days, Mean (SD) (Range)**	n=49, 266.41 (9.67) (238–284)	n=36, 272.49 (8.81) (252–287)	t-test, *p*-value=0.0038**
**Scan-motion quantification**^b^**, Mean** **(SD)**	10.20 (10.34)	8.19 (11.62)	t-test, *p*-value=0.4032
**Maternal Age at birth in years, Mean (SD) (Range)**	n=38, 36.84 (4.54) (28.6–45.6)	n=35, 34.19 (3.06) (27.3–39.8)	t-test, *p*-value=0.0049**
**Paternal Age at birth in years, Mean (SD) (Range)**	n=37, 37.71 (6.44) (23.3–56.9)	n=35, 36.15 (4.98) (28.7–46.9)	t-test, *p*-value=0.257

a defined as postnatal age at scan.

b defined as number of volumes excluded per scan. (***p-value is <0.0001, **p-value is <0.01 – 0.001, *p-value is <0.05 – 0.01).

**Table 3:** Results of univariate analyses of variance comparing DTI and NODDI parameters in the significant tracts between DS and control infants.

Tract	Parameter^a^	Down syndrome (DS)			Controls			Beta value	Lower 95%	Upper 95%	p-value	Corrected p-value	Cohen’s d
		Mean	SD	N	Mean	SD	N						

**Corpus Callosum Parietal**	AD	0.001529	3.68×10^−5^	47	0.00151	3.58×10^−5^	34	−1×10^−5^	−1.82×10^−5^	−1.74×10^−6^	0.018*	0.092	0.62
	FA	0.474409	1.93×10^−2^	47	0.46983	2.55×10^−2^	34	−2.4×10^−3^	−7.25×10^−3^	2.45×10^−3^	0.33	1	0.21
	NDI	0.309436	1.95×10^−2^	47	0.32087	2.28×10^−2^	34	6.00×10^−3^	2.04×10^−3^	0.99×10^−2^	0.0034**	0.017*	−0.55**
	ODI	0.095306	8.04×10^−3^	47	0.09903	9.29×10^−3^	34	1.76×10^−3^	−2.82×10^−4^	3.81×10^−3^	0.09	0.45	−0.43
	RD	0.00068	3.13×10^−5^	47	0.00068	3.80×10^−5^	34	−5.97×10^−7^	−7.7×10^−6^	6.5×10^−6^	0.87	1	0.07
**Corpus Callosum Tapetum**	AD	0.002154	1.2×10^−4^	46	0.00209	1.35×10^−4^	36	−3.21×10^−5^	−6.32×10^−5^	−1×10^−6^	0.043*	0.22	0.49
	FA	0.361024	2.42×10^−2^	46	0.36263	1.61×10^−2^	36	1.01×10^−6^	−5.08×10^−3^	5.08×10^−3^	0.1	1	−0.08
	NDI	0.362944	3.6×10^−2^	46	0.37466	3.15×10^−2^	36	5.56×10^−3^	−2.69×10^−3^	1.38×10^−2^	0.18	0.92	−0.34
	ODI	0.190367	3.7×10^−2^	46	0.18420	3.37×10^−2^	36	−2.66×10^−3^	−1.12×10^−2^	5.87×10^−3^	0.54	1	0.17
	RD	0.001231	1.08×10^−4^	46	0.00120	9.94×10^−5^	36	−1.56×10^−5^	−4.1×10^−5^	9.78×10^−6^	0.22	1	0.32
**CorticoSpinal Right**	AD	0.001383	4.53×10^−5^	48	0.00136	4.23×10^−5^	36	−9.87×10^−6^	−2×10^−5^	3.28×10^−7^	0.058	0.29	0.48
	FA	0.507638	1.35×10^−2^	48	0.51024	1.92×10^−2^	36	4.39×10^−4^	−3.33×10^−3^	4.21×10^−3^	0.82	1	−0.16
	NDI	0.467721	1.64×10^−2^	48	0.46948	2.17×10^−2^	36	2.47×10^−4^	−4.24×10^−3^	4.74×10^−3^	0.91	1	−0.09
	ODI	0.095056	8.91×10^−3^	48	0.09582	7.72×10^−3^	36	5.81×10^−4^	−1.42×10^−3^	2.58×10^−3^	0.57	1	−0.09
	RD	0.000576	2.75×10^−5^	48	0.00056	3.38×10^−5^	36	−7.38×10^−6^	−1.44×10^−5^	−3.73×10^−7^	0.039*	0.2	0.58
**Inferior Fronto-Occipital Fasciculus Left**	AD	0.0014	2.65×10^−5^	48	0.00142	4.63×10^−5^	34	6.54×10^−6^	−1.66×10^−6^	1.47×10^−5^	0.12	0.58	−0.43
	FA	0.395741	1.67×10^−2^	48	0.41661	1.39×10^−2^	34	9.86×10^−3^	6.27×10^−3^	1.34×10^−2^	0.00001***	0.00005***	−1.34***
	NDI	0.291474	1.35×10^−2^	48	0.30150	1.84×10^−2^	34	5.29×10^−3^	1.95×10^−3^	8.63×10^−3^	0.0023**	0.012*	−0.64**
	ODI	0.126647	9.4×10^−3^	48	0.11531	9.53×10^−3^	34	−5.27×10^−3^	−7.33×10^−3^	−3.21×10^−3^	0.00001***	0.00005***	1.20***
	RD	0.000743	2.5×10^−5^	48	0.00072	2.73×10^−5^	34	−1.15×10^−5^	−1.74×10^−5^	−5.65×10^−6^	0.0002***	0.001***	0.90***
**Inferior Fronto-Occipital Fasciculus Right**	AD	0.001407	2.7×10^−5^	45	0.00142	4.66×10^−5^	34	2.72×10^−6^	−5.77×10^−6^	1.12×10^−5^	0.53	1	−0.26
	FA	0.400235	1.69×10^−2^	45	0.41484	1.45×10^−2^	34	7.75×10^−3^	3.90×10^−3^	1.16×10^−2^	0.0001***	0.0005***	−0.92***
	NDI	0.29	1.51×10^−2^	45	0.30066	1.80×10^−2^	34	6.50×10^−3^	2.96×10^−3^	1.00×10^−2^	0.0005***	0.0025**	−0.65**
	ODI	0.120209	9.47×10^−3^	45	0.11237	1.08×10^−2^	34	−3.50×10^−3^	−5.84×10^−3^	−1.15×10^−3^	0.004**	0.02*	0.78**
	RD	0.000742	2.51×10^−5^	45	0.00072	2.61 ×10^−5^	34	−1.11×10^−5^	−1.68×10^−5^	−5.47×10^−6^	0.0002***	0.001***	0.76**
**Inferior Longitudinal Fasciculus Left**	AD	0.001502	3.31×10^−5^	35	0.00148	6.55×10^−5^	36	−1.31×10^−5^	−2.63×10^−5^	7.96×10^−8^	0.051	0.26	0.42
	FA	0.40438	2.19×10^−2^	35	0.41594	1.96×10^−2^	36	4.34×10^−3^	−9.48×10^−4^	9.64×10^−3^	0.11	0.53	−0.56
	NDI	0.276527	1.74×10^−2^	35	0.29348	2.31×10^−2^	36	8.61×10^−3^	3.81×10^−3^	1.34×10^−2^	0.0007***	0.0035**	−0.83***
	ODI	0.107502	8.26×10^−3^	35	0.10988	1.10×10^−2^	36	1.52×10^−3^	−9.92×10^−4^	4.03×10^−3^	0.23	1	−0.24
	RD	0.000781	3.28×10^−5^	35	0.00076	3.81 ×10^−5^	36	−1.02×10^−5^	−1.9×10^−5^	−1.43×10^−6^	0.024*	0.12	0.65
**Inferior Longitudinal Fasciculus Right**	AD	0.001542	5.42×10^−5^	37	0.00152	6.13×10^−5^	36	−1.1×10^−5^	−2.47×10^−5^	2.65×10^−6^	0.11	0.56	0.34
	FA	0.41299	2.4×10^−2^	37	0.42708	2.01×10^−2^	36	5.42×10^−3^	−2.28×10^−4^	1.10×10^−2^	0.06	0.3	−0.64
	NDI	0.271777	2.1×10^−2^	37	0.28676	2.22×10^−2^	36	7.46×10^−3^	2.51×10^−3^	1.24×10^−2^	0.0037**	0.019*	−0.69**
	ODI	0.104156	9.87×10^−3^	37	0.10442	8.87×10^−3^	36	9.24×10^−4^	−1.18×10^−3^	3.03×10^−3^	0.38	1	−0.03
	RD	0.000792	3.7×10^−5^	37	0.00077	3.76×10^−5^	36	−1.05×10^−5^	−1.97×10^−5^	−1.33×10^−6^	0.026*	0.13	0.66
**Superior Longitudinal Fasciculus II Left**	AD	0.001336	3.23×10^−5^	40	0.00133	4.49×10^−5^	28	−2.39×10^−6^	−1.22×10^−5^	7.44×10^−6^	0.63	1	0.10
	FA	0.332468	1.71×10^−2^	40	0.35446	2.01×10^−2^	28	1.04×10^−2^	5.77×10^−3^	1.51×10^−2^	0.00001***	0.00005***	−1.20***
	NDI	0.273937	1.56×10^−2^	40	0.28840	2.06×10^−2^	28	7.37×10^−3^	3.52×10^−3^	1.12×10^−2^	0.0003***	0.0015**	−0.81***
	ODI	0.16971	1.43×10^−2^	40	0.15322	1.49×10^−2^	28	−7.46×10^−3^	−1.14×10^−2^	−3.51×10^−3^	0.0004***	0.002**	1.13***
	RD	0.000798	2.81×10^−5^	40	0.00077	3.44×10^−5^	28	−1.48×10^−5^	−2.2×10^−5^	−7.54×10^−6^	0.0001***	0.0005***	1.00***
**Superior Longitudinal Fasciculus II Right**	AD	0.001328	3.23×10^−5^	33	0.00133	4.42×10^−5^	33	−1.64×10^−6^	−1.12×10^−5^	7.95×10^−6^	0.73	1	0.04
	FA	0.339356	2.07×10^−2^	33	0.35405	1.66×10^−2^	33	6.85×10^−3^	1.99×10^−3^	1.17×10^−2^	0.0064**	0.032*	−0.78**
	NDI	0.279004	1.67×10^−2^	33	0.29318	1.95×10^−2^	33	7.38×10^−3^	3.59×10^−3^	1.11×10^−2^	0.0002***	0.001***	−0.78**
	ODI	0.160886	1.53×10^−2^	33	0.15268	1.35×10^−2^	33	−3.81×10^−3^	−7.62×10^−3^	−7.68×10^−6^	0.05	0.25	0.57
	RD	0.000778	3.02×10^−5^	33	0.00076	3.05×10^−5^	33	−1×10^−5^	−1.7×10^−5^	−2.93×10^−6^	0.0062**	0.031*	0.64**
**Uncinate Fasciculus Left**	AD	0.001386	2.94×10^−5^	39	0.00140	3.59×10^−5^	35	3.53×10^−6^	−4.61×10^−6^	1.17×10^−5^	0.39	1	−0.32
	FA	0.370956	1.65×10^−2^	39	0.38330	1.35×10^−2^	35	5.31×10^−3^	1.57×10^−3^	9.05×10^−3^	0.006**	0.03*	−0.81***
	NDI	0.271155	1.57×10^−2^	39	0.27838	1.80×10^−2^	35	3.77×10^−3^	−2×10^−4^	7.75×10^−3^	0.062	0.31	−0.43
	ODI	0.147031	1.17×10^−2^	39	0.13870	1.06×10^−2^	35	−3.09×10^−3^	−5.88×10^−3^	−3.09×10^−4^	0.03*	0.15	0.74
	RD	0.000772	2.52×10^−5^	39	0.00076	2.55×10^−5^	35	−6.35×10^−6^	−1.23×10^−5^	−4.04×10^−7^	0.037*	0.18	0.53
**Uncinate Fasciculus Right**	AD	0.001389	2.92×10^−5^	40	0.00141	3.80×10^−5^	36	9.25×10^−6^	1.01×10^−6^	1.75×10^−5^	0.028*	0.14	−0.62
	FA	0.360311	1.41×10^−2^	40	0.37634	1.45×10^−2^	36	6.95×10^−3^	3.61×10^−3^	1.02×10^−2^	0.00001***	0.00005***	−1.12***
	NDI	0.263935	1.51×10^−2^	40	0.26904	1.79×10^−2^	36	2.44×10^−3^	−1.42×10^−3^	6.31×10^−3^	0.21	1	−0.31
	ODI	0.14925	1.1×10^−2^	40	0.13852	1.07×10^−2^	36	−4.07×10^−3^	−6.74×10^−3^	−1.40×10^−3^	0.0033**	0.017*	0.99***
	RD	0.000785	2.56×10^−5^	40	0.00077	2.78×10^−5^	36	−6.81×10^−6^	−1.29×10^−5^	−7.22×10^−7^	0.029*	0.15	0.61

a FA, NDI, and ODI are unitless values representing a range in these respective properties between 0 and 1. AD, and RD are reported in mm^2^/s. (***p-value is ≤0.001, **p-value is ≤0.01, *p-value is <0.05; Cohen’s d: *small effect is 0.2 – 0.5, **medium effect is 0.5 – 0.8, ***large effect is ≥ 0.8).

Abbreviations: AD, axial diffusivity; FA, fractional anisotropy; NDI, neurite density index; ODI, orientation dispersion index; RD, radial diffusivity.

## Data Availability

Data will be made available on request.
