# Supplementary material for: Early White Matter Microstructure Alterations in Infants with Down Syndrome
Source: Neuroimage. Author manuscript; Available in PMC 2025 Nov 19. (PMC12629210; doi:10.1016/j.neuroimage.2025.121489)
Supplement: 1 [file NIHMS2121403-supplement-1.docx]

**Supplementary Material**

**Methods**

**IBIS Infant Studies Background:**

The study is part of the Infant Brain Imaging Study (IBIS), an ongoing multisite longitudinal study, collecting developmental behavioral and neuroimaging data on infants with neurodevelopmental conditions. The IBIS–DS studies infants with and without DS between the ages of 6 and 24 months. The IBIS–Early Prediction (IBIS-EP) studies infants at elevated familial likelihood for ASD between the ages of 6 and 24 months. Data collection occurred at five sites: The Children’s Hospital of Philadelphia (CHOP), The University of Minnesota (UMN), The University of North Carolina at Chapel Hill (UNC), The University of Washington (UW), and Washington University in St. Louis (WashU). Infants were recruited, scanned, and examined between March 2019 and May 2024.

**Exclusion Criteria:**

Exclusion criteria for all infants included: a diagnosis or physical signs of known genetic conditions (other than DS in DS infants), significant medical conditions the could impact growth, development, cognition, or sensory function (except for conditions commonly associated with DS in DS infants), birth weight <2500 grams, gestational age <34 weeks for DS infants or <36 weeks for control infants, history of significant perinatal complications, prenatal exposure to neurotoxins, maternal gestational diabetes requiring medication, contraindication for MRI, and families whose primary language is not English, due to requirements for cognitive assessments.

**Along-tract analyses:**

Each tract was resampled into equally spaced nodes along its arc length, with a uniform step size of 1, enabling standardized node-wise comparisons across participants. Since analyses were limited to diffusion parameters (FA, RD, AD, NDI, ODI) that showed significant group differences in prior ANOVA tests, no additional correction for multiple comparisons was applied. Group comparisons were covaried by age at assessment (in days), sex, and scan-motion quantification. A significance threshold of p < 0.05 was used to identify region-specific differences, defined as localized segments along the tract showing consistent differences across adjacent nodes.

**
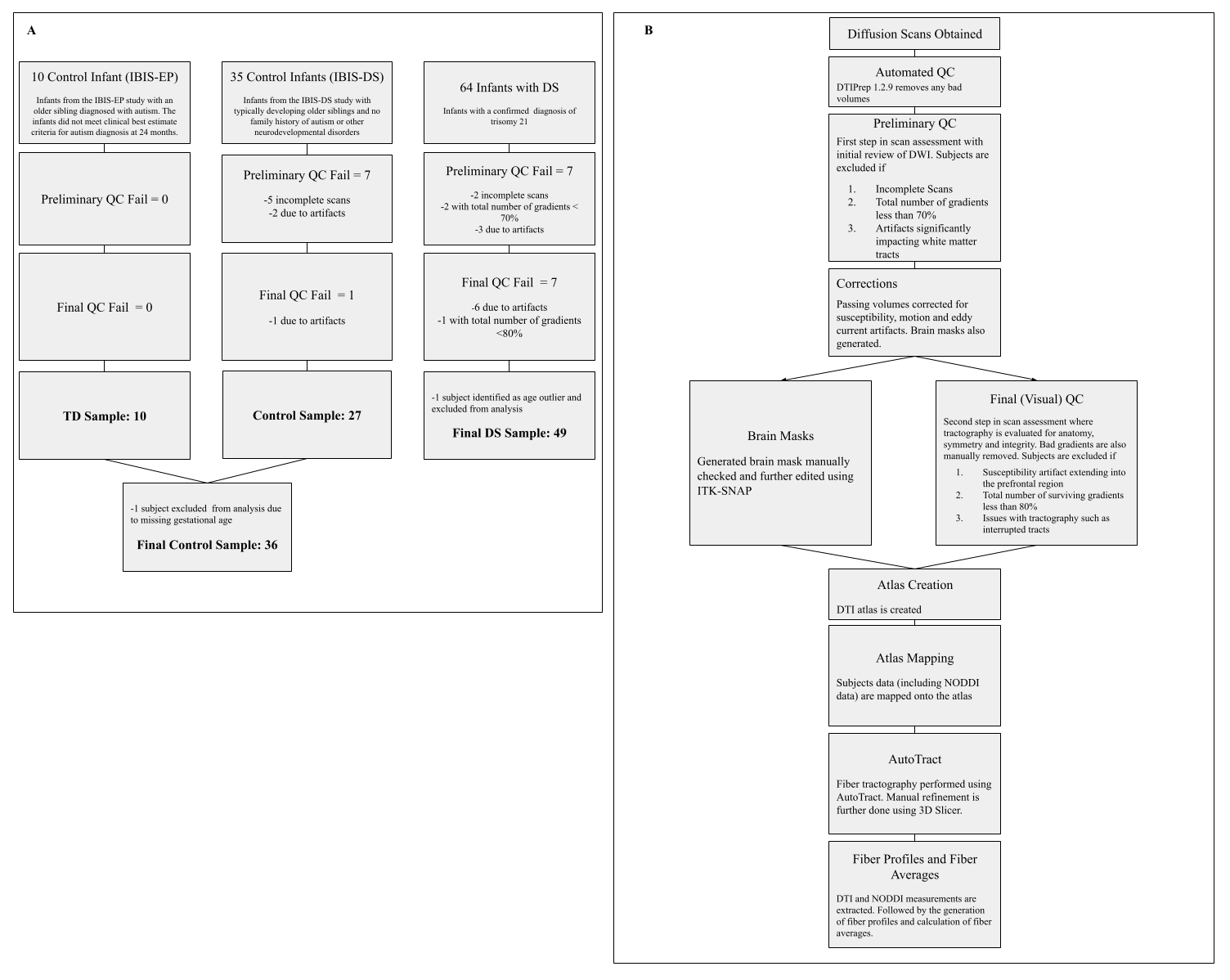
Supplementary Figure 1. (A) Flow diagram showing the number of participants excluded at each stage of data processing. (B) Visual overview of the full data processing pipeline.** No significant group differences were found in the number of excluded scans (*p*=0.5998).

**Supplementary Figure 2. An example of the susceptibility artifact is seen bilaterally in the temporal poles on high b-value (A) and low b-value (B) DWI.**


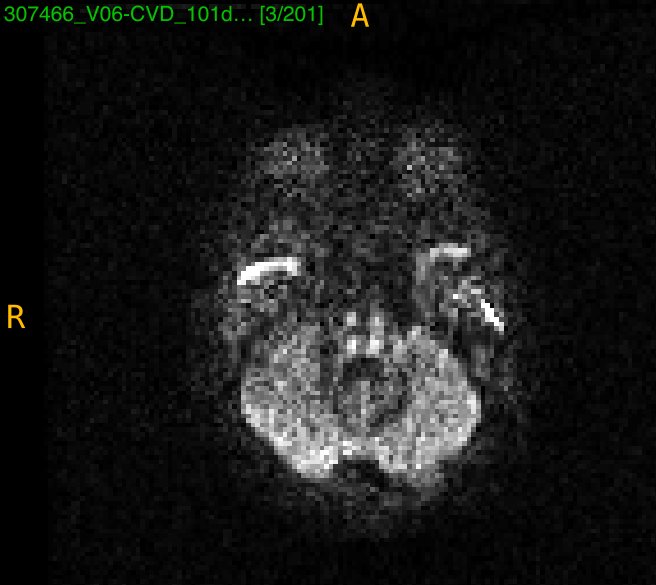

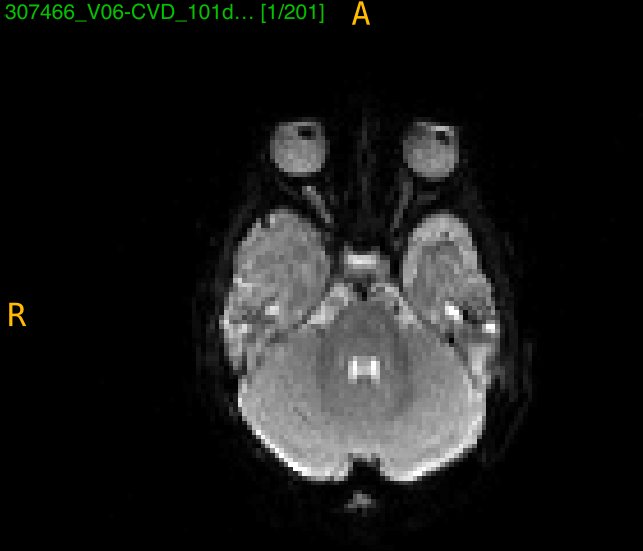


A

B

| Interhemispheric Tracts | Intrahemispheric Tracts^a^ |
| --- | --- |
| Corpus Callosum Body (CC Body) | Arcuate Fasciculus FrontoParietal |
| Corpus Callosum Genu (CC Genu) | Arcuate Fasciculus FrontoTemporal |
| Corpus Callosum Motor (CC Motor) | Arcuate Fasciculus TemporoParietal |
| Corpus Callosum Parietal (CC Parietal)* | Cingulate Gyrus part of the Cingulum (CGC) |
| Corpus Callosum PreMotor (CC PreMotor) | Corticofugal Motor |
| Corpus Callosum Splenium (CC Splenium)* | Corticofugal Parietal |
| Corpus Callosum Tapetum (CC Tapetum)* | Corticofugal PreFrontal* |
|  | Corticofugal PreMotor |
|  | CorticoSpinal Tract (CST)* |
|  | Corticothalamic Motor |
|  | Corticothalamic Parietal |
|  | Corticothalamic PreFrontal |
|  | Corticothalamic PreMotor |
|  | Corticothalamic Superior |
|  | Fornix |
|  | Hippocampal part of the Cingulum (CGH) |
|  | Inferior Fronto-Occipital Fasciculus (IFOF)* |
|  | Inferior Longitudinal Fasciculus (ILF)* |
|  | Optic Radiation |
|  | Optic Tract |
|  | Superior Longitudinal Fasciculus II (SLF II)* |
|  | Uncinate Fasciculus (UNC)* |

**Supplementary Table 1. Index of all tracts examined in exploratory analyses.** ^a^indicates bilateral tracts analyzed separately for the left and right hemispheres. ^*^indicates tracts included in the primary analyses.

|  | *Down syndrome (DS)* | *Controls* | *Group Comparison* |
| --- | --- | --- | --- |
| **Maternal Education, Mean (SD)** | 4.24 (1.30) | 4.65 (1.15) | t-test, *p*-value=0.1636 |
| 1. Some high school | 0 (0%) | 0 (0%) |  |
| 2. High school graduate | 3 (6.12%) | 0 (0%) |  |
| 3. Some college | 8 (16.33%) | 5 (13.89%) |  |
| 4. College graduate | 15 (30.61%) | 15 (41.67%) |  |
| 5. Some grad school | 1 (2.04%) | 1 (2.78%) |  |
| 6. Graduate degree | 11 (22.45%) | 13 (36.11%) |  |
| NA. Not available | 11 (22.45%) | 2 (5.56%) |  |
| **Paternal Education, Mean (SD)** | 4.027 (1.4237) | 4.514 (1.5024) | t-test, *p*-value=0.1621 |
| 1. Some high school | 0 (0%) | 0 (0%) |  |
| 2. High school graduate | 7 (14.29%) | 4 (11.11%) |  |
| 3. Some college | 5 (10.2%) | 6 (16.67%) |  |
| 4. College graduate | 15 (30.61%) | 9 (25%) |  |
| 5. Some grad school | 0 (0%) | 0 (0%) |  |
| 6. Graduate degree | 10 (20.41%) | 16 (44.44%) |  |
| NA. Not available | 12 (24.49%) | 1 (2.78%) |  |
| **Household Income, Mean (SD)** | 5.8 (1.86) | 6.09 (1.38) | t-test, *p*-value=0.4680 |
| 1. less than 25K | 2 (4.08%) | 0 (0%) |  |
| 2. 25K-35K | 0 (0%) | 0 (0%) |  |
| 3. 35K-50K | 1 (2.04%) | 1 (2.78%) |  |
| 4. 50K-75K | 6 (12.25%) | 4 (11.11%) |  |
| 5. 75K-100K | 2 (4.08%) | 6 (16.67%) |  |
| 6. 100K-150K | 11 (22.45%) | 11 (30.56%) |  |
| 7. 150K-200K | 6 (12.25%) | 6 (16.67%) |  |
| 8. over-200K | 7 (14.29%) | 7 (19.44%) |  |
| NA. Not available | 14 (28.57%) | 1 (2.78%) |  |

**Supplementary Table 2. Education and household income by group.** (^***^*p*-value is ≤0.001, ^**^*p*-value is ≤0.01, ^*^*p*-value is <0.05).

| Tract | Test | Multivariate Analysis of Variance | | | | Cohort | |
| --- | --- | --- | --- | --- | --- | --- | --- |
|  |  |  |  |  |  | Exact F | *p*-value |
|  |  | Value | Approx. F | *p*-value | Corrected *p*-value |  |  |
| **Corpus Callosum Parietal** | Wilks' Lambda | 0.297285 | 4.0974 | 0.00001*** | 0.00015*** | 6.8326 | 0.00001*** |
|  | Pillai's Trace | 0.936255 | 3.4559 | 0.00001*** | 0.00015*** |  |  |
| **Corpus Callosum Splenium** | Wilks' Lambda | 0.435475 | 2.7356 | 0.00001*** | 0.00015*** | 1.408 | 0.2315 |
|  | Pillai's Trace | 0.682579 | 2.4347 | 0.0002*** | 0.003** |  |  |
| **Corpus Callosum Tapetum** | Wilks' Lambda | 0.498826 | 2.2152 | 0.001*** | 0.015* | 4.1981 | 0.0021** |
|  | Pillai's Trace | 0.584147 | 2.0107 | 0.0031** | 0.0465* |  |  |
| **Corticofugal PreFrontal Left** | Wilks' Lambda | 0.516019 | 2.1843 | 0.0012** | 0.018* | 1.8437 | 0.1146 |
|  | Pillai's Trace | 0.596401 | 2.1399 | 0.0013** | 0.0195* |  |  |
| **Corticofugal PreFrontal Right** | Wilks' Lambda | 0.542652 | 2.0041 | 0.0038** | 0.057 | 1.6123 | 0.1672 |
|  | Pillai's Trace | 0.550362 | 1.9543 | 0.0044** | 0.066 |  |  |
| **Corticospinal Left** | Wilks' Lambda | 0.551244 | 1.9484 | 0.0053** | 0.0795 | 4.1035 | 0.0024** |
|  | Pillai's Trace | 0.519602 | 1.8324 | 0.0093** | 0.1395 |  |  |
| **Corticospinal Right** | Wilks' Lambda | 0.53481 | 2.0287 | 0.0033** | 0.0495* | 4.2115 | 0.002** |
|  | Pillai's Trace | 0.553453 | 1.9417 | 0.0048** | 0.072 |  |  |
| **Inferior Fronto-Occipital Fasciculus Left** | Wilks' Lambda | 0.279263 | 4.4079 | 0.00001*** | 0.00015*** | 8.2384 | 0.00001*** |
|  | Pillai's Trace | 0.979035 | 3.7009 | 0.00001*** | 0.00015*** |  |  |
| **Inferior Fronto-Occipital Fasciculus Right** | Wilks' Lambda | 0.401323 | 2.8732 | 0.00001*** | 0.00015*** | 4.8944 | 0.0007*** |
|  | Pillai's Trace | 0.751661 | 2.5832 | 0.00001*** | 0.00015*** |  |  |
| **Inferior Longitudinal Fasciculus Left** | Wilks' Lambda | 0.422594 | 2.381 | 0.0004*** | 0.006** | 4.0351 | 0.0031** |
|  | Pillai's Trace | 0.726493 | 2.21 | 0.0009*** | 0.0135* |  |  |
| **Inferior Longitudinal Fasciculus Right** | Wilks' Lambda | 0.360821 | 2.9748 | 0.00001*** | 0.00015*** | 3.8627 | 0.0041** |
|  | Pillai's Trace | 0.852064 | 2.7526 | 0.00001*** | 0.00015*** |  |  |
| **Superior Longitudinal Fasciculus II Left** | Wilks' Lambda | 0.196067 | 4.7777 | 0.00001*** | 0.00015*** | 9.0889 | 0.00001*** |
|  | Pillai's Trace | 1.185779 | 3.855 | 0.00001*** | 0.00015*** |  |  |
| **Superior Longitudinal Fasciculus II Right** | Wilks' Lambda | 0.27165 | 3.5221 | 0.00001*** | 0.00015*** | 7.9692 | 0.00001*** |
|  | Pillai's Trace | 0.889807 | 2.5979 | 0.00001*** | 0.00015*** |  |  |
| **Uncinate Fasciculus Left** | Wilks' Lambda | 0.448448 | 2.306 | 0.0007*** | 0.0105* | 2.4053 | 0.0462* |
|  | Pillai's Trace | 0.674893 | 2.1222 | 0.0016** | 0.024* |  |  |
| **Uncinate Fasciculus Right** | Wilks' Lambda | 0.321184 | 3.5287 | 0.00001*** | 0.00015*** | 3.9910 | 0.0032** |
|  | Pillai's Trace | 0.940557 | 3.2437 | 0.00001*** | 0.00015*** |  |  |

**Supplementary Table 3. Results of Multivariate analyses of variance comparing DTI and NODDI parameters in the tracts of interest between DS and control infants.** (^***^*p*-value is ≤0.001, ^**^*p*-value is ≤0.01, ^*^*p*-value is <0.05).
